# Effect of *ALDH2 rs671* and *ADH1B rs1229984* polymorphisms on habitual alcohol use: evidence from a large Taiwanese cohort

**DOI:** 10.1192/bjo.2026.12042

**Published:** 2026-07-21

**Authors:** Mu-En Liu, Yu-Ting Yan, Yu-Tung Tien, Wei-Ming Liu, Ding-Lieh Liao, Po-Hsiu Kuo, Shih-Jen Tsai

**Affiliations:** Department of Psychiatry, Taipei Veterans General Hospital, Taipei, Taiwan; Department of Psychiatry, College of Medicine, National Yang Ming Chiao Tung University, Taipei, Taiwan; Department of Public Health & Institute of Epidemiology and Preventive Medicine, National Taiwan University, Taipei, Taiwan; Department of General Psychiatry, Yuli Hospital, Hualien City, Taiwan; Department of Psychiatry, Taoyuan Psychiatric Center, Taoyuan City, Taiwan; Psychiatric Research Center, Wan Fang Hospital, Taipei Medical University, Taipei, Taiwan

**Keywords:** Alcohol, *ADH1B*, *ALDH2*, genetic polymorphism, odds ratio

## Abstract

**Background:**

Two functional genetic polymorphisms, *ADH1B rs1229984* and *ALDH2 rs671*, play key roles in ethanol metabolism and are especially prevalent in East Asian populations. The *rs1229984* variant accelerates the conversion of ethanol to acetaldehyde, whereas *rs671* reduces enzymatic activity for converting acetaldehyde into acetic acid. These variants affect alcohol use disorder risk and have been implicated in various systemic conditions.

**Aims:**

To investigate the broader associations of *ADH1B rs1229984* and *ALDH2 rs671* with habitual alcohol use in large, unselected populations.

**Method:**

We analysed data from 146 374 Taiwanese adults (aged 20–90 years) enrolled in the Taiwan Biobank.

**Results:**

Both variants showed clear associations with habitual alcohol use. The *ADH1B rs1229984* CC genotype was associated with a higher prevalence of drinking, indicating a substantial contribution to alcohol-related behaviour. The *ALDH2 rs671* A allele was linked to markedly reduced drinking, reflecting known intolerance among carriers rather than implying a uniformly larger effect than *ADH1B*. Beyond alcohol use, *rs671* showed a protective association with gout, whereas *rs1229984* was not significantly associated with chronic diseases after correction.

**Conclusions:**

In this large population sample, both *ADH1B rs1229984* and *ALDH2 rs671* demonstrate meaningful, genotype-dependent effects on habitual alcohol use. Their combined effects underscore the importance of evaluating gene–gene interactions rather than attributing disproportionate influence to a single locus.

Alcohol metabolism is a key indicator of an individual’s susceptibility to a diverse range of metabolic, cardiovascular, hepatic and neuropsychiatric conditions.^1^ Two well-characterised functional genetic variants involved in alcohol metabolism are *rs1229984* in the alcohol dehydrogenase 1B (*ADH1B*) gene and *rs671* in the aldehyde dehydrogenase 2 (*ALDH2*) gene.^2^ These variants are particularly prevalent in East Asian populations and affect the activities of enzymes involved in ethanol metabolism.^2^

## *ADH1B rs1229984* and *ALDH2 rs671* variants

The *ADH1B rs1229984* T (or ‘A’) allele produces a hyperactive alcohol dehydrogenase-1B enzyme that oxidises ethanol to acetaldehyde 20- to 40-fold faster than the ancestral C (or ‘G’) allele.^3^ High acetaldehyde levels cause unpleasant symptoms, such as facial flushing, nausea and rapid heartbeat, which discourage alcohol consumption. Thus, individuals with the *rs1229984* T allele tend to drink less and are substantially less likely to develop alcohol use disorder (AUD).^4^ This protective effect has been widely observed in various cultures, particularly among East Asians, where the allele is more common. Both candidate gene and genome-wide association studies have reported that the *rs1229984* T allele is strongly associated with a decreased risk of AUD.^5–7^ The *ALDH2 rs671* variant results from a G-to-A transition and an amino acid substitution of lysine for glutamic acid. The G and A alleles encode the active and inactive forms of *ALDH2*, respectively. Because of decreased *ALDH2* activity in people with the A allele, the oxidation of acetaldehyde to acetic acid is markedly impaired.^8^ After alcohol consumption, individuals with the *rs671* AA genotype have higher blood acetaldehyde levels than do those with the GG genotype and thus experience facial flushing, tachycardia and nausea. These aversive reactions make AA homozygotes particularly intolerant to alcohol and reduce their risk of AUD.^4,9^

## Physiological functions of *ADH1B* and *ALDH2*

*ADH1B* and *ALDH2* serve physiological functions beyond alcohol metabolism, particularly in endogenous aldehyde detoxification, retinoid metabolism and redox homeostasis, processes that affect metabolic, cardiovascular, oncologic and neurological health.^10–13^ In addition to AUD, previous studies have identified associations between these two genetic variants and various health outcomes. For example, individuals with the *rs1229984* T allele have lower systolic blood pressure, interleukin-6 levels, waist circumference and body mass index, as well as decreased odds of coronary heart disease and ischaemic stroke.^12–14^ In European cohorts, *rs1229984* is associated with gout and a high uric acid level, but only among those who consume alcohol. Among non-drinkers, this association is not observed, suggesting a gene–environment interaction instead of a direct genetic effect.^15^

The extent to which these two genetic variants of alcohol-metabolising enzymes affect habitual alcohol use in large, unselected populations remains insufficiently characterised. Thus, in the present study, we investigated the associations of *rs1229984* and *rs671* with habitual alcohol use in a Taiwanese cohort. Using data from over 145 000 participants, we investigated the effects of these variants on the risks of habitual alcohol use and common chronic conditions, including gout and cardiovascular disorders. All analyses were adjusted for demographic and lifestyle factors and accounted for population stratification by using principal components. The large sample size and high prevalence of these genetic variants in the study population offered a unique opportunity to explore their systemic effects on human health.

## Method

### Participants

The study cohort comprised 146 374 Taiwanese participants from the Taiwan Biobank.^16–19^ Between 2013 and 2022, the Taiwan Biobank recruited individuals from Taiwan and collected biological specimens and related data through multiple recruitment sites.^18,19^ Supported primarily by the Taiwanese Government, the Taiwan Biobank aims to advance scientific research on major public health topics and promote collaboration among researchers studying prevalent chronic diseases in the region.^17,20,21^ Eligible participants were aged 20 years or older, self-identified as being of Taiwanese ancestry at the time of recruitment and able to perform daily activities independently.^19^

The authors assert that all procedures contributing to this work comply with the ethical standards of the relevant national and institutional committees on human experimentation and with the Helsinki Declaration of 1975, as revised in 2013. All procedures involving human participants were approved by the Institutional Review Board of Taipei Veterans General Hospital (approval number: 2023-04-007CC#1). All participants provided written informed consent at recruitment.

### Measures

Disease status was determined using a self-reported questionnaire. For most clinical conditions, the concordance between self-reports in the Taiwan Biobank and Taiwan’s National Health Insurance claims records ranged from moderate to excellent.^17^ Participants were classified as having type 2 diabetes if their fasting blood glucose was ≥126 mg/dL or their haemoglobin A1c was ≥6.5%, regardless of self-reported status. Current smoking was defined as regular smoking for at least 6 months and ongoing smoking at the time of evaluation. Habitual alcohol use was defined as consumption of more than 150 mL of alcohol per week for at least 6 months and continued use at the time of evaluation.

### Genotyping

DNA was extracted from blood samples by using the QIAamp DNA blood kit following the manufacturer’s instructions (Qiagen, Valencia, California, USA). Single-nucleotide polymorphism (SNP) genotyping was conducted using the Axiom Genome-Wide Array Plate System (Affymetrix, Santa Clara, California, USA) with custom Taiwan Biobank chips. The SNP panel included the *ADH1B rs1229984* and *ALDH2 rs671* polymorphisms.

### Statistics

After imputation, 650 000 independent SNPs were used to perform principal component analysis, and the first 10 principal components were calculated for each individual. Logistic regression models were used to determine the relationships between the SNPs and self-reported current smoking or alcohol use, after adjustments for age, self-reported gender and the first ten principal components. To examine associations between the two SNPs and self-reported diseases, logistic regression models were employed with adjustments for age, self-reported gender, habitual alcohol use, current smoking and the first ten principal components. Only diseases with a prevalence >1% in the population were included in the logistic regression analysis. Odds ratios were calculated based on the number of effect alleles, and Bonferroni correction was applied to control for multiple comparisons.

Associations between biochemical markers and the two SNPs were analysed by linear regression models with the same covariates. In the biochemical analyses, data points beyond the mean ± 3 s.d. were considered outliers and excluded. Bonferroni correction was applied to control for multiple testing. SNPs were coded as 0, 1 or 2 in accordance with the number of minor alleles present.

## Results

This study investigated the associations of the genetic variants *rs1229984* and *rs671* with various clinical and biochemical characteristics, as well as habitual alcohol use, by using data from a large Taiwanese cohort. A total of 146 374 individuals (53 006 men and 93 368 women) aged 20–90 years (mean 49.42, s.d. 11.38 years) were included in the analysis. Adjustments were made for age, gender, current smoking and drinking status and genetic population structure. The genotype distributions of *rs1229984* and *rs671* were consistent with Hardy–Weinberg equilibrium (*P* = 0.38 and 0.75, respectively).

**Fig. 1:**
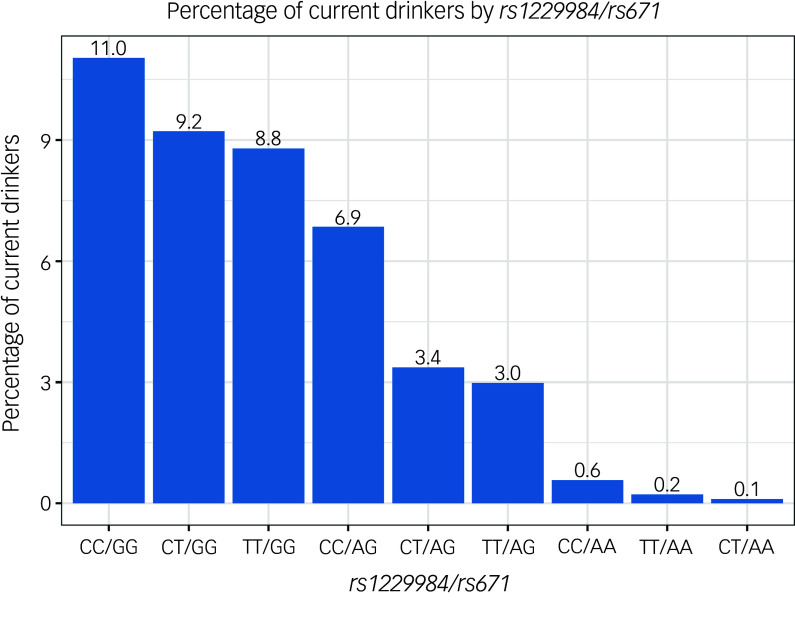
The percentage of individuals with habitual alcohol use, stratified by nine genotype combinations of two polymorphisms: *ADH1B rs1229984* and *ALDH2 rs671*.

Table 1 presents the associations between the genetic polymorphisms *rs1229984* and *rs671* and current smoking and habitual alcohol use. For *rs1229984*, individuals with the CC genotype (8.50%) had significantly higher rates of habitual alcohol consumption than those with the TT genotype (5.74%), with an odds ratio of 1.19 and a highly significant *P*-value (1.88E−22), indicating a strong association between this SNP and habitual alcohol use. By contrast, *rs1229984* was not significantly associated with smoking behaviour (odds ratio 1.00, *P* = 0.740). For *rs671*, individuals with the AA genotype had markedly low rates of habitual alcohol use (0.20%) and a substantially low odds ratio of 0.29 (*P <* 1E−300), indicating that this genotype effectively protected against alcohol consumption. Figure 1 illustrates the percentage of individuals with habitual alcohol use, stratified by nine genotype combinations of two polymorphisms: *ADH1B rs1229984* and *ALDH2 rs671*. The data show a clear trend in which alcohol consumption rates vary significantly depending on these genetic profiles. The highest percentage of current drinkers (11.0%) is observed in individuals with the CC genotype at *rs1229984* and GG genotype at *rs671*; i.e. those with slow alcohol metabolism and fully active *ALDH2*. In contrast, the lowest percentages (0.1–0.2%) are seen in individuals with the AA genotype at *rs671* (inactive *ALDH2*), especially when paired with CT or TT genotypes at *rs1229984*. Intermediate drinking rates are observed in individuals with heterozygous genotypes (AG at *rs671*), indicating a dose-dependent effect of *ALDH2* enzymatic activity on drinking behaviour. The data support a synergistic interaction: both high-activity *ADH1B* (TT or CT) and low-activity *ALDH2* (AA) contribute independently and additively to reducing alcohol consumption. Furthermore, we performed a *post hoc* comparison of *ADH1B rs1229984* CT versus TT genotypes across all *ALDH2* strata. The CT genotype showed a modest but statistically significant higher rate of habitual drinking than TT (6.1 *v*. 5.7%, odds ratio 1.07, *P* = 3.20E–04), consistent with the semidominant enzymatic activity pattern. No significant association was noted between *rs671* and current smoking (odds ratio 1.00, *P* = 0.98) (Table 1).

**Table 1:** Analysis of current smoking and habitual alcohol use in 146 374 participants in accordance with the *ADH1B rs1229984* and *ALDH2 rs671* genotypes

Variable	*rs1229984*					*rs671*				
Genotype (minor allele number)	CC (2)	CT (1)	TT (0)	Odds ratio	*P*-value	AA (2)	AG (1)	GG (0)	Odds ratio	*P*-value
Genotype number	10 352	56 902	79 120			11 752	59 358	75 264		
Current smoker (%)	9.48	9.30	9.48	1.00	7.40E–01	9.26	9.47	9.39	1.00	9.76E–01
Habitual alcohol use (%)	8.50	6.12	5.74	1.19	**1.88E–22**	0.20	3.40	9.11	0.29	**<1E–300**

Logistic regression models were used to examine the relationship between genotype and current smoking or habitual alcohol use after adjusting for age, gender and the first ten principal components. *P*-values in bold are statistically significant after Bonferroni correction for multiple comparisons (0.05/2 = 0.025).

We investigated the associations between the genetic variants *rs1229984* and *rs671* and various chronic diseases (Table 2). Logistic regression models were adjusted for age, self-reported gender, current smoking, drinking status and genetic population structure. Among the associations tested, the *rs671* A allele had a statistically significant protective effect against gout. Individuals with the A allele (AA or AG genotypes) of *rs671* had significantly lower odds of gout (odds ratio 0.85). Table 3 presents gene–environment interaction analyses demonstrating that *ALDH2 rs671* exerts a protective association with gout that is only modestly modified by drinking status. In both the dominant and additive models, *rs671* shows a significant main effect (odds ratios of approximately 0.83–0.86) with extremely small *P*-values, indicating a robust reduction in gout risk independent of covariates. Current drinking is associated with higher gout risk (odds ratios of approximately 1.23–1.25). The *rs671*×drinking interaction reaches nominal significance only in the additive model (*P* = 0.047) and is nonsignificant in the dominant model, suggesting that interaction effects are present, but relatively small. Stratified analyses reveal that the *rs671* protective effect is stronger among current drinkers (odds ratio 0.71), and still clearly present among non-drinkers (odds ratio 0.86), demonstrating that the variant reduces gout risk both with and without alcohol exposure, but the magnitude of protection is enhanced in individuals who drink. Stratified analyses indicate that the *ALDH2 rs671* variant is protective against gout in both drinkers and non-drinkers, but with different magnitudes of effect. Although models adjusted for habitual alcohol use and stratified analyses were performed, alcohol consumption may lie on the causal pathway between *ALDH2* genotype and gout, making covariate adjustment difficult to interpret causally. Future studies with quantitative alcohol intake data and longitudinal outcome ascertainment are needed to clarify whether the association reflects mediation through alcohol consumption, effect modification by alcohol exposure or alcohol-independent biological mechanisms. Among current drinkers, carriers of the A allele exhibit a markedly reduced gout risk (odds ratio 0.71), with a significant *P*-value (2.60E−04), indicating stronger genetic protection when alcohol exposure is present. Among non-drinkers, the protective effect remains significant, but is more modest (odds ratio 0.86, *P* = 8.98E−10).

**Table 2:** Analysis of physical and mental diseases based on dominant genetic models for *ADH1B rs1229984* (CC versus CT/TT) and *ALDH2 rs671* (GG versus AG/AA) in 146 374 participants Table 2 long description.

Genotype	*rs1229984*				*rs671*			
	CC (1)	CT/TT (0)	Odds ratio	*P*-value	GG (1)	AA/AG (0)	Odds ratio	*P*-value
Gout (%)	3.69	3.81	1.00	9.54E–01	4.14	3.44	1.23	**2.23E–12**
Asthma (%)	4.13	3.67	1.12	3.12E–02	3.69	3.70	0.99	7.13E–01
Emphysema bronchitis (%)	1.02	1.13	0.89	2.55E–01	1.15	1.09	1.04	4.02E–01
Valve heart disease (%)	4.18	4.23	0.97	5.74E–01	4.30	4.16	1.02	3.77E–01
Coronary artery disease (%)	1.28	1.31	0.99	9.22E–01	1.29	1.34	0.97	5.33E–01
Arrhythmia (%)	4.64	4.47	1.03	5.56E–01	4.60	4.36	1.04	1.25E–01
Hyperlipidaemia (%)	7.30	7.54	0.96	3.66E–01	7.66	7.39	1.04	7.61E–02
Hypertension (%)	11.79	12.17	0.97	3.20E–01	12.29	12.00	1.01	4.24E–01
Peptic ulcer (%)	14.23	14.22	1.01	7.84E–01	14.22	14.23	1.01	7.23E–01
Gastroesophageal reflux (%)	14.39	14.12	1.01	6.34E–01	14.22	14.05	1.00	9.75E–01
Irritable bowel syndrome (%)	2.68	2.53	1.06	3.51E–01	2.60	2.47	1.06	1.12E–01
Depression (%)	3.79	3.67	1.01	8.13E–01	3.68	3.69	0.99	6.32E–01
Migraine (%)	2.80	2.92	0.94	2.96E–01	2.83	2.99	0.94	4.80E–02
Hepatobiliary stone (%)	4.48	4.51	1.00	9.66E–01	4.49	4.52	0.99	8.43E–01
Kidney stone (%)	6.51	6.30	1.06	1.88E–01	6.32	6.30	1.01	5.35E–01
Type 2 diabetes mellitus (%)	8.71	9.65	0.90	6.79E–03	9.46	9.71	0.98	3.02E–01

Employing logistic regression models to examine the relationship between clinical conditions and genotypes, controlling for age, gender, current smoking, habitual alcohol use and the first ten main principal components. *P*-values in bold are statistically significant after corrections for multiple comparisons (0.05/16 = 0.003125).

**Table 3:** Interaction effects of the *rs671* genotype and current drinking on gout

Predictor	Odds ratio	*P*-value
*rs671* (dominant model)	0.83	**5.01E−10**
Current drinking	1.23	**1.51E−05**
*rs671*×drinking interaction	0.87	1.61E−01
*rs671*, per effect allele (additive model)	0.86	**7.18E−10**
Current drinking	1.25	**4.20E−06**
*rs671*×drinking interaction	0.83	**4.69E−02**

Model adjusted for age, gender, current smoking and the first ten principal components, with gout as the outcome, *rs671* and current drinking as predictors, and an *rs671* × DRK_ current drinking term. *P*-values in bold are statistically significant after corrections for multiple comparisons.

By contrast, *rs1229984* was not associated with any disease after correction for multiple testing. Overall, these findings indicate a strong and specific inverse association between the *rs671* A allele and gout. No other diseases, including cardiovascular, neuropsychiatric or gastrointestinal disorders, were significantly associated with either variant after adjustment for multiple comparisons.

## Discussion

The present study used data from more than 145 000 individuals to comprehensively evaluate habitual alcohol use and the systemic health effects of 2 genetic polymorphisms, *rs1229984* in *ADH1B* and *rs671* in *ALDH2*, in a Taiwanese population cohort. The findings provide robust evidence linking these variants to alcohol consumption behaviours (Table 1 and Fig. 1). The *rs1229984* variant, located in the *ADH1B* gene, affects alcohol metabolism. Individuals with the CC genotype had a significantly higher prevalence of habitual drinking, as indicated by an odds ratio of 1.19 and a significant *P*-value (*P* = 1.88E–22) (Table 1). This finding suggests that genetic variation at this locus affects alcohol tolerance and preference. The rs671 *variant* in the *ALDH2* gene demonstrated an even more pronounced effect: individuals with the AA genotype had a considerably low prevalence of habitual alcohol use (0.20%), with a *P*-value approaching zero (<1E−300), indicating a markedly strong association. Figure 1 illustrates the combined effects of *ALDH2 rs671* and *ADH1B rs1229984* genotypes on habitual alcohol consumption. Individuals with the AA genotype at *rs671* (inactive *ALDH2*) have the lowest percentages, particularly when combined with the CT or TT genotypes at *rs1229984*, whereas those with the *rs671* GG and *rs1229984* CC genotypes had the highest prevalence (11.0%) (Fig. 1). Liver tissue activity for *ADH1B rs1229984* CC, CT and TT has been reported as 1:5:6, with the T allele being semidominant.^8^ Numerous prior Asian investigations investigated the risk disparity in alcohol dependence or drinking behaviours between CT and TT genotypes; nevertheless, significant differences were frequently absent because of the minimal variation in enzyme activity. An analysis of this magnitude should reveal a highly significant difference, even with a minor risk disparity between CT and TT, regardless of overall results or *ALDH2* genotype. These genotype combinations strongly affect drinking behaviour. Notably, neither variant was associated with smoking behaviour (odds ratios near 1 and nonsignificant *P*-values) (Table 1), indicating that their genetic effect is specific to alcohol use instead of general substance use. These interactions highlight a biologically plausible mechanism: individuals who both produce acetaldehyde quickly (via the *ADH1B* T allele) and cannot clear it effectively (via the *ALDH2* A allele) are most likely to avoid alcohol. In public health, this genetic information helps create models to predict habitual alcohol use, especially in East Asian populations, where these genetic variants are often found. It also underscores the importance of considering gene–gene interactions rather than evaluating single variants in isolation when assessing behavioural phenotypes. These results are consistent with the findings of a previous study indicating that these genetic variants contribute to AUD susceptibility^4^ and further demonstrate their influence on habitual alcohol use. The observed associations have implications for public health strategies and the development of personalised interventions targeting alcohol-related health risks.

Among the diseases examined, *rs671* exhibited a significant protective effect against gout, with an odds ratio of 0.85 and a significant *P*-value (Table 2). This association remained robust after applying Bonferroni correction and is consistent with the findings of previous studies linking *rs671* to gout risk.^22,23^ Further stratified analyses indicate that the *ALDH2 rs671* variant is protective against gout in both drinkers and non-drinkers. These results demonstrate that *rs671* lowers gout susceptibility regardless of drinking status, whereas alcohol consumption amplifies the protective impact of the variant, even after adjustment for age, gender, smoking and population structure.

Although *rs671* was previously reported to be associated with hypertension,^24,25^ type 2 diabetes mellitus^25^ and coronary artery disease,^26^ these associations were not observed in the present study. The discrepancy may stem from differences in sample composition, such as alcohol-using cohorts versus community-based samples, or variations in diagnostic criteria, including reliance on clinical diagnoses, hospital records or biomarker thresholds.

*rs1229984* demonstrated limited associations in the present study. Although we observed suggestive trends for triglyceride levels and red blood cell count, none reached Bonferroni-corrected significance. These findings may be attributed to the relatively small systemic impact of this variant beyond its well-established role in acute alcohol metabolism.

### Research and clinical implications

The genetic effect of *rs671* and *rs1229984* on habitual alcohol use highlights several avenues for further investigation. The interactions between these polymorphisms and lifestyle or dietary factors should be systematically explored. Furthermore, the integration of polygenic risk scores with *ALDH2* and *ADH1B* genotypes may enhance risk prediction for alcohol-related diseases in East Asian populations.

### Limitations

The primary strength of this study lies in its large, ethnically homogeneous sample, which provides high statistical power and reduces the risk of population stratification confounding. However, this study has several limitations that should be considered. Medical diagnoses were based on self-reported data, which are inherently subject to recall bias, misclassification and social desirability bias.^27^ Habitual alcohol use was defined using a binary threshold based on the Taiwan Biobank questionnaire, which cannot capture dose–response gradients among drinkers. The lack of detailed quantitative consumption data precluded more refined analyses of drinking intensity, beverage type, cumulative alcohol exposure or lifetime drinking trajectories. As a result, genotype-specific differences in alcohol dose among drinkers could not be evaluated, and may have attenuated estimates of gene–environment interaction effects. In addition, behavioural data, particularly alcohol consumption, may not accurately capture lifetime exposure. Future longitudinal studies incorporating biomarker validation of alcohol intake and disease progression are needed to better elucidate causal relationships. Finally, relying solely on participants from the Taiwan Biobank may not precisely reflect the actual impact of these two SNPs on health. Selection bias may arise as the cohort comprised self-selected volunteers, who are typically more health-conscious and predisposed to healthier lives.

## Data Availability

The data that support the findings of this study are available from Taiwan Biobank Institutional Dataset. To gain access, interested individuals should contact ‘biobank@gate.sinica.edu.tw’.

## Table 2 Long description

A table comparing the associations between genetic variants rs1229984 and rs671 and various chronic diseases. The table has 18 rows and 10 columns. The columns are labeled as follows: Genotype, rs1229984 CC (1), rs1229984 CT/TT (0), rs1229984 Odds ratio, rs1229984 P-value, rs671 GG (1), rs671 AA/AG (0), rs671 Odds ratio, rs671 P-value. The rows are labeled with different chronic diseases and conditions. Each row presents the percentage values for each genotype, odds ratio, and P-value for both genetic variants. The table includes data for conditions such as gout, asthma, emphysema bronchitis, valve heart disease, coronary artery disease, arrhythmia, hyperlipidemia, hypertension, peptic ulcer, gastroesophageal reflux, irritable bowel syndrome, depression, migraine, hepatobiliary stone, kidney stone, and type 2 diabetes mellitus.

Navigate back to Table 2.
